# Effect of Early Interdisciplinary Rehabilitation for Trauma Patients: A Systematic Review

**DOI:** 10.1016/j.arrct.2020.100070

**Published:** 2020-06-25

**Authors:** Hanne Langseth Naess, Eirik Vikane, Eike Ines Wehling, Jan Sture Skouen, Rae Frances Bell, Lars Gunnar Johnsen

**Affiliations:** a Regional Trauma Center, Haukeland University Hospital, Bergen, Norway; b Department of Physical Medicine and Rehabilitation, Haukeland University Hospital, Bergen, Norway; c Department of Biological and Medicine Psychology, University of Bergen, Bergen, Norway; d Department of Global Public Health and Primary Care, University of Bergen, Bergen, Norway; e Regional Centre of Excellence in Palliative Care, Haukeland University Hospital, Bergen, Norway; f Department of Neuromedicine and Movement Science, University of Trondheim, Trondheim, Norway; g Norwegian National Advisory Unit on Trauma, Oslo, Norway

**Keywords:** Patient reported outcome measures, Rehabilitation, Trauma, nervous system, Wounds and injuries, EIR, early interdisciplinary rehabilitation, ICU, intensive care unit, RCT, randomized controlled trial, TBI, traumatic brain injury, WHO, World Health Organization

## Abstract

**Objective:**

To perform a systematic review to assess the current scientific evidence concerning the effect of EIR for trauma patients with or without an associated traumatic brain injury.

**Data Source:**

We performed a systematic search of several electronic (Ovid MEDLINE, Embase, Cochrane Library Central Register of Controlled Trials, Cumulative Index to Nursing and Allied Health, and SveMed+) and 2 clinical trial registers (clinicaltrials.gov and International Clinical Trials Registry Platform). In addition, we handsearched reference lists from relevant studies.

**Data Extraction:**

Two review authors independently identified studies that were eligible for inclusion. The primary outcome measures were functional-related outcomes and return to work. The secondary outcome measures were length of stay in hospital, number of days on respirator, complication rate, physical and mental health measures, quality of life, and socioeconomic costs.

**Data Synthesis:**

Four studies with a total number of 409 subjects, all with traumatic brain–associated injuries, were included in this review. The included trials varied considerably in study design, inclusion and exclusion criteria, and had small numbers of participants. All studies were judged to have at least 1 high risk of bias. We found the quality of evidence, for both our primary and secondary outcomes, low.

**Conclusions:**

No studies that matched our inclusion criteria for EIR for trauma patients without traumatic brain injuries could be found. For traumatic brain injuries, there are a limited number of studies demonstrating that EIR has a positive effect on functional outcomes and socioeconomic costs. This review highlights the need for further research in trauma care regarding early phase interdisciplinary rehabilitation.

Studies on rehabilitation in cerebral stroke and spinal cord injuries show that early interdisciplinary rehabilitation (EIR) and a continued chain of rehabilitation accelerate the rate of recovery and improve functional outcomes.^1^^,^^2^ Furthermore, a continuous chain of rehabilitation is cost-effective.^2^, ^3^, ^4^, ^5^ A systematic review^6^ on multidisciplinary rehabilitation intervention in trauma survivors published in 2011 found a lack of high-quality studies and was unable to conclude whether multidisciplinary rehabilitation for this population is effective or not.

The primary model for collaboration in most hospitals is one where the different professionals work in parallel, in accordance with each one’s profession-specific goal and treatment plan, with the aim of accomplishing discipline-specific goals. In an interdisciplinary model the team members work together, both in treatment and goal setting.^7^^,^^8^ In EIR, the early onset of rehabilitation interventions is essential to prevent complications and promote recovery. Interdisciplinary rehabilitation comprises team-based interventions using the International Classification of Functioning, Disability, and Health model alongside curative, supportive, preventive, and palliative strategies.^9^

EIR is poorly defined in the literature. In studies concerning severe traumatic brain injury (TBI), “early” means within hours or few days, with the intervention started in the intensive care unit. A patient with TBI admitted to a trauma hospital without a specific EIR program will typically, in addition to medical care, get conventional physical therapy, occupational therapy, speech therapy, and help from a social worker concerning psychosocial issues. There seems to be consensus on early rehabilitation interventions for this specific trauma population, although the approach is not always interdisciplinary. Even though EIR is the preferred approach within rehabilitation medicine for all severely injured patients, with the exception of conventional physical therapy, early interventions for trauma patients without associated head injury seem to be lacking.

Major trauma refers to physical injury or a combination of injuries where there is a strong possibility of death or disability and is commonly defined using an Injury Severity Score^10^ threshold of 15.

During the past decades acute trauma care has improved because of the development of highly specialized trauma centers and teams. As a result, the mortality rate for severely injured patients has decreased.^11^ However, motor vehicle crashes alone are responsible for 1.35 million deaths per year worldwide and is the leading cause of death for children and young adults.^12^ Exact numbers of cases of disability due to trauma injuries are lacking. The European Commission estimates that about 135,000 persons experience serious injury due to traffic accidents each year.^13^ Because trauma patients are often young, the traumatic event may result in lifelong physical, cognitive, and emotional limitations that compromise an independent, self-determined life. Trauma patients report reduced quality of life, pain problems, and anxiety or depression several years after the traumatic event.^14^ Trauma is also associated with considerable socioeconomic costs related to return to work and use of disability benefits.^15^

This review examines the effect of EIR for trauma patients. To our knowledge, no previous systematic review has addressed this topic.

## Methods

### Protocol and registration

The review protocol was registered in PROSPERO, an International Prospective Register of Systematic Reviews at the National Institute for Health Research and Centre of Reviews and Dissemination at the University of York (registration no. CRD42018111541).

### Types of studies

We considered the following studies to be eligible for inclusion:

• Randomized controlled trials (RCTs)
• Observational studies with a comparative group
• Prospective longitudinal before and after studies
• Health economic evaluation studies

### Study selection

#### Inclusion criteria

*Population:* Studies including adult patients admitted to the hospital with injuries following trauma. In our protocol we define adult as 18 years or older, which is the legal adulthood in most countries. Because most studies examining an adult population include participants from 16 years of age, we decided to deviate from the originally registered protocol and to include studies including adult participants 16 years and older.

*Intervention group:* Trauma patients receiving early interdisciplinary rehabilitation. “Early” was defined as within 1 week of admission to hospital because of acute traumatic injury. “Rehabilitation” was defined according to the World Health Organization (WHO) definition: “a set of measures that assist individuals who experience, or are likely to experience, disability to achieve and maintain optimal functioning in interaction with their environments” (World report on disability, Chapter 4, page 96).^16^

*Control group:* The control group received treatment other than EIR.

*Outcome measures:* The primary outcome measures were (1) functional outcome (eg, FIM, Barthel Index for activities of daily living, Glasgow Outcome Scale, Glasgow Outcome Scale Extended, Cognitive Function Scale); and (2) return to work. Secondary outcomes were (1) length of stay in the intensive care unit (ICU), (2) total length of stay in hospital, (3) complication rate, (4) physical health, (5) mental health, (6) quality of life, and (7) socioeconomic costs.

#### Exclusion criteria

Because of confusion in the vocabulary and in the literature concerning terms describing interdisciplinary teams or rehabilitation teams, we assessed all studies where early rehabilitation was described in the abstract and excluded those that addressed intervention from a single profession. Articles describing an integrated early interdisciplinary rehabilitation without a comparison group and single case studies were also excluded.

### Search strategy

To identify studies concerning early interdisciplinary rehabilitation for trauma patients, a research librarian searched the following electronic databases: US National Library of Medicine/PubMed (from 1946 to June 2018); Embase (from 1974 to June 2018); Cochrane Library (Wiley); Cumulative Index to Nursing and Allied Health (EVSCO) (from 1981 to June 2018); and SveMed+. Clinical trial registers (clinicaltrials.gov) and the WHO International Clinical Trials Registry Platform were searched for unpublished data.

Further search strategies included handsearching of reference lists from systematic reviews and relevant studies, and gray matter relevant for this topic.

The search was conducted in June 2018 and an updated search was performed in July 2019. The key search terms used to identify potential studies are listed in supplemental appendix S1 (available online only at http://www.archives-pmr.org/).

### Data collection and analysis

#### Selection of studies

Studies identified by the electronic searches were registered in EndNoteX9.a After removal of duplicates, titles were exported to Rayyan,^17^ a web application for systematic reviews. The titles and abstracts of all studies were then independently screened by 2 reviewers (H.L.N., E.V.) for potential relevance regarding the inclusion and exclusion criteria (step 1). Full-text copies of the articles identified in step 1 were then retrieved and assessed for eligibility (step 2).

Any disagreement was resolved by discussion and, if necessary, by consulting with a third reviewer (J.S.).

#### Data extraction and management

The first author (H.L.N.) extracted data from the included studies and entered them into a text-based form. The data were then controlled and discussed with a second reviewer (E.V.). The summary/consensus from each trial is listed in supplemental appendix S2 (available online only at http://www.archives-pmr.org/).

#### Assessment of the risk of bias

To assess the quality of the included studies we used the Cochrane risk-of-bias tool for randomized trials.^18^

Quality was assessed by 2 reviewers (H.L.N., E.V.). Disagreements were resolved by discussion. Consensus was reached without the need to consult with a third reviewer.

## Results

### Results of the search

**Fig 1:**
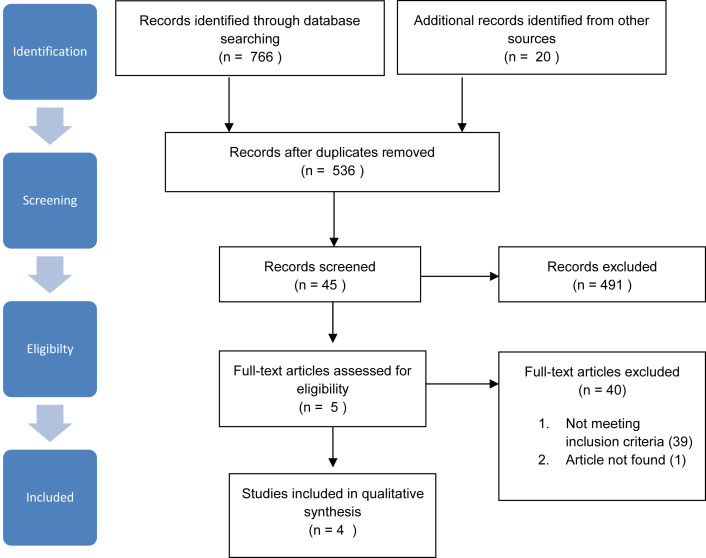
PRISMA flow diagram detailing search strategy and selection criteria.; Abbreviation: PRISMA, Preferred Reporting Items for Systematic Reviews and Meta-Analyses.

The searches identified 766 titles: 239 from PubMed, 336 from Embase, 20 from the Cochrane library, 157 from the Cumulative Index to Nursing and Allied Health, and 14 from SveMed+. Nine titles were identified from Clinicaltrials.gov and 1 from WHO: International Clinical Trials Registry Platform. An additional 10 titles were identified from reference lists. After removal of duplicates, 536 titles were screened for assessment. A total of 491 titles were excluded, and 45 titles were included after the first screening. After the second screening, 40 articles were excluded and 5 were assessed for eligibility. Two publications concerned the same study. Four studies were included in this review. A summary/consensus from the second screening is listed in supplemental appendix S3 (available online only at http://www.archives-pmr.org/). Search strategy and selection is shown in figure 1.

### Included studies

We assessed 5 full-text articles.^3^^,^^19^, ^20^, ^21^, ^22^ Because 2 of the publications^3^^,^^19^ concerned the same study, only 4 studies were included in this review. The studies varied considerably in design and inclusion/exclusion criteria. All participants had TBI. One study addressed mild TBIs^20^; the remainder addressed severe TBIs. Only 1 of the 4 studies was an RCT.^20^ The characteristics of the included studies are listed in supplemental appendix S2. All studies were judged to have at least 1 high risk of bias. Risk of bias summary is listed in table 1.

**Table 1:** Risk of bias summary

Study	Random Sequence Generation	Allocation Concealment	Blinding of Participants and Personnel	Blinding of Outcome Assessment	Incomplete Outcome Data: Objective Outcomes	Incomplete Outcome Data: Subjective Outcomes	Selective Reporting	Other Bias
Andelic et al^3^^,^^19^	High	High	High	High	Low	Low	Low	Low
Ghaffar et al^20^	Low	Unclear	High	Unclear	High	High	Low	Low
Lui et al^21^	High	High	High	High	Low	Low	Low	Low
Mackay et al^22^	High	High	High	Unclear	Low	Low	Low	High

### Efficacy

#### Primary outcomes

Few studies reported any of our primary outcomes. An overview of primary outcomes is shown in table 2.

**Table 2:** Overview primary outcomes

Measures	Andelic et al^3^^,^^19^	Ghaffar et al^20^	Lui et al^21^	Mackay et al^22^
GOSE	x			
FIM			x	
DRS	x			
RLA				x
Cognitive test		x		
Return to work	x			

Abbreviations: DRS, Disability Rating Scale; GOSE, Glasgow Outcome Scale Extended; RLA, Rancho Los Amigos Scale of Cognitive Functioning.

Andelic et al^3^^,^^19^ included 61 subjects, 31 in the intervention group and 30 in the control group. They found a favorable functional outcome at 12 months and at 5 years post injury for participants receiving EIR and a continuous chain of rehabilitation compared with participants with a broken rehabilitation chain.

Mackay et al^22^ included 38 subjects, 17 in the intervention group and 21 in the control group. They found a positive effect of an EIR intervention on cognitive function at discharge from the acute ward and on discharge from a rehabilitation unit compared with a historical control group (previously treated patients).

In the 5-year follow-up study by Andelic^3^ 59 of the 61 subjects from the 2012 study^19^ were included. The study examined, among other things, return to work. The authors found no statistical difference in this outcome between the intervention and control groups.

#### Quality of studies primary outcomes

None of these studies were RCTs. Therefore, they were judged to have a moderate to high risk of bias. There were few participants in the trials, making the results uncertain. For the primary outcome we assessed the quality of the evidence to be low.

#### Secondary outcomes

Few studies reported on our secondary outcomes. An overview of secondary outcomes is shown in table 3.

**Table 3:** Overview secondary outcomes

Variables	Andelic et al^3^^,^^19^	Ghaffar et al^20^	Lui et al^21^	Mackay et al^22^
LOS ICU	x		x	
LOSt	x		x	x
Complication rate			x	
HADS				
SF-36				
GHQ		x		
Socioeconomic costs	x			

Abbreviations: GHQ, General Health Questionnaire; HADS, Hospital Anxiety and Depression Scale; LOS, length of stay; LOSt, length of stay total; SF-36, Short Form-36 Health Survey.

Andelic et al^19^ reported the length of stay in the ICU. The median stay in the ICU for the intervention group was 12 days vs 13.5 days in the control group. This difference was not statistically significant.

Three studies reported on the length of stay in total.^19^^,^^21^^,^^22^ Although Mackay^22^ noted a trend toward a favorable outcome in the intervention group, the difference did not reach statistical significance. In the trial by Lui et al^21^ that included 119 subjects, 68 in the intervention group and 51 in the control group, there was no statistically significant difference between groups regarding the total length of stay, as was the case for the trial by Andelic et al.^19^

Lui^21^ also examined complications as an outcome. They could not find a statistically significant difference between the intervention and control groups for this outcome.

Ghaffar et al^20^ did an RCT where they included 191 subjects, 97 in the intervention group and 94 in the control group. The study reported on health-related quality of life. They could not find a significant difference between intervention and control groups_._

Andelic^19^ reported on socioeconomic costs. They found a favorable result for the intervention group, although the difference was not significant.

#### Quality of studies secondary outcomes

Only 1 study^20^ was an RCT that reported on health-related quality of life, with the risk of bias considered to be moderate. None of the other studies were RCTs. The risk of bias was considered to be moderate to high. With few participants the results are uncertain. For the secondary outcomes we assessed the quality of the evidence to be low.

Table 4 shows a summary of results for efficacy outcomes from predefined outcomes in individual studies. Further details are listed in supplemental appendix S4 (available online only at http://www.archives-pmr.org/).

**Table 4:** Summary of efficacy outcomes in individual studies

Study	Outcome	Effect Size
Andelic et al^3^^,^^19^	GOSE	*P*=.03
	DRS	*P*=.03
	Return to work	NS
	LOS ICU	NS
	LOS total	NS
	DRS	NS
	Total hospitalization costs at 1 y	NS
	Total hospitalization costs at 5 y	NS
Ghaffar et al^20^	Cognitive tests	NS
Lui et al^21^	FIM	NS
	LOS total	NS
	Complications during hospital stay	NS
Mackay et al^22^	RLA acute	*P*=.003
	RLA rehabilitation	*P*=.05
	LOS total	*P*=.028

Abbreviations: DRS, Disability Rating Scale; GOSE, Glasgow Outcome Scale; LOS, length of stay; NS, not significant; RLA, Rancho Los Amigos Scale of Cognitive Functioning.

#### Type of intervention

Three of the included studies^19^^,^^21^^,^^22^ examined the effect of EIR in an inpatient setting, while 1 trial^20^ examined the effect of EIR for mild TBI in an outpatient setting_._

In the trial by Andelic^19^ participants in the intervention group received treatment from an interdisciplinary team comprising an early rehabilitation program based on Affolter (organization of sensory input), Bobath (stimulation of normal movement, function, and control), and Coombs (retraining functions of the face and mouth). Participants received a mean of 105 minutes of treatment daily. When medically stable, the participants in the EIR group were directly transferred to an inpatient rehabilitation unit. The control group received either inpatient brain injury rehabilitation in a subacute rehabilitation department after a waiting period at a local hospital or nursing home or received no inpatient rehabilitation. In the subacute phase of TBI, all patients received a daily minimum of 2-3 hours of individual treatment including physiotherapy, occupational therapy, speech therapy, cognitive training, nutrition, dietary services, and psychosocial support.

In the study by Lui et al^21^ the rehabilitation team included a physiatrist, nurse, physiotherapist, occupational therapist, speech therapist, dietician, and medical social worker. The patients were screened within 72 hours of admission and included in the study if they had a TBI diagnosis. Participants in the study received 30-120 minutes therapy per day, 5 days per week, starting in the acute neurosurgery unit.

The physiatrist-led team had twice-weekly multidisciplinary reviews including a rehabilitation plan where they also determined the need for further inpatient rehabilitation. Participants who were considered in need of further inpatient rehabilitation were transferred to the acute inpatient rehabilitation unit or to subacute rehabilitation facilities at the local community hospital. The need for further inpatient rehabilitation was determined according to best available literature on known factors predicting recovery after TBI, country, social- and cultural-specific data, and multidisciplinary team consensus meetings. Medical data from former patients were used as a comparison. The data comprised patients with TBI who received inpatient rehabilitation in the same acute rehabilitation unit prior to implementation of the early integrated TBI rehabilitation program. The patients in the control group were usually referred to rehabilitation by the department of neurosurgery.

Mackay et al^22^ did a retrospective review in which they examined the medical record of participants who were discharged from an inpatient rehabilitation facility between 1984 and 1990. Seventeen of these received acute services at a hospital with a formalized early intervention TBI program, while the control group comprised 21 participants who received acute care services at 10 different hospitals that did not have a formalized early intervention TBI program. The formalized early intervention TBI program focused on cooperation between trauma and rehabilitation services (formalized biweekly meetings), trauma rehabilitation, team approach treatment, information for and involvement of family members, and discharge planning by team members and family. The trauma rehabilitation involved evaluation and treatment by a physiatrist, physical therapist, occupational therapist, and speech and language therapist following admission to the hospital. The study intervention involved structured multisensory stimulation, orientation, exercise, and positioning to prevent contractures and sensory deprivation.^22^

The RCT by Ghaffar et al^20^ included participants with mild TBI. The intervention group was given an appointment at a multidisciplinary TBI clinic within 1 week of injury. Participants and their spouse or a relative were educated by an occupational therapist in a standardized manner, with reference to a checklist of postconcussion symptoms and possible effects of mild TBI. At each visit, participants were assessed by an occupational therapist, a neurorehabilitation physician, and a neuropsychiatrist. Treatment was tailored according to the individual patient`s needs and included pharmacotherapy, supportive psychotherapy, physiotherapy, and occupational therapy. Follow-up visits varied in frequency from weekly to bimonthly. The participants in the control group were discharged from hospital without treatment or follow-up visits.

None of the studies presented a decision tree to guide the team in their decision to plan the patient rehabilitation trajectory.

#### Harm and adverse events

No study reported specifically on harm or serious adverse events.

#### Withdrawals

No withdrawals because of adverse events or lack of efficacy were reported.

## Discussion

The aim of this study was to perform a systematic review to assess the current scientific evidence concerning EIR for trauma patients. No studies that matched our inclusion criteria for early rehabilitation for trauma patients without TBIs could be found. Four studies were included in this review, all concerning EIR for TBI. The included studies varied considerably in design and inclusion and exclusion criteria. There were small numbers of participants, different outcome measures, and 3 of 4 studies were not randomized. None of the studies reported on harm or adverse events.

Andelic et al^19^ found that 81% of participants receiving EIR were living at home, and 13% were in a nursing home at 12 months post injury. In the control group only 53% were living at home. Furthermore, they found that a greater percentage of participants in the EIR group returned to work (part or full time). The results are of clinical importance for patients with severe TBI. However, the study is not an RCT, and it is the only study in this review that examines return to work after trauma. Using the GRADE criteria we found the results of this study to be of low quality. The lack of blinding and few patients is a common problem when studying the effect of rehabilitation after severe injuries, and some authors have suggested an adapted scoring system in this field.^23^^,^^24^

Apart from the study by Ghaffar et al,^20^ all participants in the included studies received some form of rehabilitation. The main difference between the intervention and care-as-usual groups was the time from injury to the start of rehabilitation. With low numbers of participants and similar treatment in both intervention and control groups, the sensitivity of these trials was compromised. The likelihood of detecting a difference between the interventions, had there been one, was low with the outcome measures used. The lack of a standardized outcome assessment battery is another major problem when studying the effects of EIR. The development of specific and sensitive outcome measures for this heterogeneous group of patients is an important goal because many factors may confound the treatment effects in these studies.^24^^,^^25^

EIR has primary focus on collaboration between departments and direct transfer to a rehabilitation unit as soon as the patient is medically stable. The formalized cooperation between the acute ward and the rehabilitation unit seems to be beneficial for faster transfer to rehabilitation and reduction in the length of stay in the acute ward. Mackay et al^22^ found that trauma patients with head injuries who received early rehabilitation (formalized TBI program) during acute hospitalization had a shorter length of stay both in the acute ward and the rehabilitation ward and significantly higher cognitive levels at discharge compared with trauma patients who were treated in hospitals that did not have a formalized TBI program. Bouman et al^26^ investigated the effect of fast track rehabilitation for all types of injuries. This fast track rehabilitation included coordination of treatment by the trauma surgeon and the rehabilitation physician. This formalized cooperation resulted in an earlier transfer to the rehabilitation unit and a tendency to better functional outcomes in the fast track group at 6-month follow-up. The study did not examine early-onset (within 7d) interdisciplinary rehabilitation and was therefore not found eligible for further investigation in this review.

The clinical implication for the individual trauma patient with a shorter stay in an acute ward is the potential positive effect of optimizing resources within the hospital (available beds in acute wards) and also a potential beneficial economic effect. One of the included studies examined the effect on medical complications for patients receiving EIR.^21^ Lui et al found a lower rate of medical complications for patients receiving EIR than the control group not receiving EIR. The difference was not statistically significant but may be of clinical importance. Medical complications are common in trauma patients and have a negative effect on outcome, increase mortality, and prolong hospital stay.^27^^,^^28^ Whyte et al^28^ found a reduction of medical complications in relation to time in rehabilitation. The authors concluded that increased medical stability was related to the length of the brain injury rehabilitation, not time since the injury. In 1 of the included studies delay in in-hospital rehabilitation was shown to have a negative effect on functional outcomes and a negative outcome on socioeconomic costs.^19^ These results are all interesting and support the importance of EIR.

Questions such as who should be offered EIR, when to start, and what type of EIR intervention has the greatest effect on functional outcomes in the trauma population remain unanswered and are important topics for future clinical research. Follow-up studies from trauma populations have found that trauma patients report pain, anxiety, depression, reduced quality of life, and reduced work ability several years after the trauma.^14^^,^^15^ Whether or not EIR can have a beneficial effect on these outcomes is as yet unknown. A formalized cooperation between trauma and rehabilitation units followed by direct transfer to a rehabilitation unit as soon as the patient is medically stable may be beneficial for better functional outcomes.^19^^,^^21^^,^^26^

Evidence from high-quality studies of patients with stroke and spinal injury indicates that EIR has an important effect on outcomes.^1^^,^^2^ While this review is unable to provide clear support for benefit for EIR, it does not provide clear evidence of a lack of benefit.

However, there is a clear need for further research using standardized interventions and outcomes and larger numbers of participants. Random assignment, which has been successfully used regularly in trials for more than 50 years, is the preferred method.^29^ When looking for comparison studies, we hoped to find RCTs where the efficacy of the EIR model was compared with treatment as usual, in other words, the current best standard of care. This method is the best way of getting sufficient reliable data, but unfortunately, no such studies were identified. Achieving sufficient power in an RCT in this setting is a challenge, and it may be unethical to replace standardized treatment with alternative treatment as a control. A qualitative study focusing on patient needs with the aim of developing a core outcome set for future RCTs could also be of value. In this regard, studies comparing practices in welfare systems in different countries could also be of interest.

### Study limitations

A major limitation is the lack of a uniform definition for early rehabilitation in the literature. In this review “early” was defined as within 1 week of admission to hospital because of acute traumatic injury. The definition of “early phase” might have been too narrow to include all studies investigating rehabilitation in trauma patients. In particular studies including severely injured trauma patients with prolonged stay in intensive care units are missing. However, because of the definition, our findings demonstrate the lack of consensus about when to start early rehabilitation interventions.

## Conclusions

There is currently no strong evidence that EIR is more effective than care as usual for TBIs. Methodological issues need to be addressed. There is a clear need of high-quality trials on EIR using standardized interventions and outcomes and including larger numbers of participants.

For trauma patients without TBI we could not find studies that matched our inclusion criteria for EIR. This highlights the need for research in trauma care regarding EIR for all patients with major trauma injuries.

Further research on EIR vs standard care could potentially influence trajectories in the early phase after trauma. In the near future, we hope rehabilitation will have the same central position and focus in traumatology as acute care.

## Supplier

a. EndNote Version X9; Clarivate Analytics.
