# Supplementary material for: Effect of Early Interdisciplinary Rehabilitation for Trauma Patients: A Systematic Review
Source: Arch Rehabil Res Clin Transl. 2020 Jun 25;2(4):100070. doi: 10.1016/j.arrct.2020.100070 (PMC7853396; doi:10.1016/j.arrct.2020.100070)
Supplement: Supplemental Appendix S1 - S4 [file mmc1.docx]

**Supplemental Appendix S1** – electronic search 26.06.18, key search terms

Database: Ovid MEDLINE(R) <1946 to Present>

1 exp Multiple Trauma/ (12068)

2 exp Trauma, Nervous System/ (191451)

3 (multiple trauma or polytrauma or multitrauma or multi-trauma or (trauma* adj3 (spinal cord or nervous system or brain or amputation*))).ti,ab,kw. (44379)

4 1 or 2 or 3 (216421)

5 exp REHABILITATION/ (274032)

6 rehabilitation.fs. (183993)

7 rehabilitation.ti,ab,kw. (141652)

8 5 or 6 or 7 (467696)

9 (early or acute or fast track).ti,ab,kw. (2295342)

10 4 and 8 and 9 (3833)

11 exp Patient Care Team/ (63428)

12 ((multi?disciplinary or inter?disciplinary or integrated or multi?modal or multi?professional) adj3 (therap* or restor* or care* or team* or rehab*)).ab,ti,kw. (43371)

13 11 or 12 (99430)

14 10 and 13 (222)

# Database: Embase (OVID) <1974 to present>

1 multiple trauma/ (13083)

2 exp nervous system injury/ (296057)

3 (multiple trauma or polytrauma or multitrauma or multi-trauma or (trauma* adj3 (spinal cord or nervous system or brain or amputation*))).ti,ab,kw. (64978)

4 1 or 2 or 3 (318776)

5 exp rehabilitation/ (346844)

6 rh.fs. (147821)

7 rehabilitation.ti,ab,kw. (205632)

8 5 or 6 or 7 (531988)

9 (early or acute or fast track).ti,ab,kw. (3108576)

10 4 and 8 and 9 (7059)

11 ((multi?disciplinary or inter?disciplinary or integrated or multi?modal or multi?professional) adj3 (therap* or restor* or care* or team* or rehab*)).ab,ti,kw. (70298)

12 10 and 11 (313)

# Database: Cochrane library (Wiley)

#1 MeSH descriptor: [Multiple Trauma] explode all trees 241

#2 MeSH descriptor: [Trauma, Nervous System] explode all trees 4748

#3 "multiple trauma" or polytrauma or multitrauma or multi-trauma or "multi trauma":ti,ab,kw (Word variations have been searched) 513

#4 trauma* near/3 ("spinal cord" or "nervous system" or brain or amputation*):ti,ab,kw (Word variations have been searched) 3090

#5 #1 or #2 or #3 or #4 6896

#6 MeSH descriptor: [Rehabilitation] explode all trees 31762

#7 rehabilitation:ti,ab,kw (Word variations have been searched) 25553

#8 #6 or #7 50867

#9 early or acute or "fast track":ti,ab,kw (Word variations have been searched) 191779

#10 #5 and #8 and #9 263

#11 (multi-disciplinary or multidisciplinary or inter-disciplinary or interdisciplinary or integrated or multi-modal or multimodal or multi-professional or multiprofessional) near/3 (therap* or restor* or care* or team* or rehab*):ti,ab,kw (Word variations have been searched) 4925

#12 #10 and #11 13

# Database: CINAHL (EBSCO), 1981- nå

S1 (MH "Multiple Trauma") 1,803

S2 (MH "Spinal Cord Injuries+") OR (MH "Head Injuries+") OR (MH "Amputation, Traumatic") OR (MH "Spinal Injuries+") 45,934

S3 TI ( multiple trauma or polytrauma or multitrauma or multi-trauma or (trauma* N2 (spinal cord or nervous system or brain or amputation*)) ) OR AB ( multiple trauma or polytrauma or multitrauma or multi-trauma or (trauma* N2 (spinal cord or nervous system or brain or amputation*)) ) 12,284

S4 S1 OR S2 OR S3 49,553

S5 (MH "Rehabilitation+") 186,442

S6 TI Rehabilitation OR AB Rehabilitation 57,923

S7 S5 OR S6 216,635

S8 TI ( early or acute or "fast track" ) OR AB ( early or acute or "fast track" ) 237,263

S9 S4 AND S7 AND S8 1,906

S10 (MH "Multidisciplinary Care Team+") 28,437

S11 TI ( (multi#disciplinary or inter#disciplinary or integrated or multi#modal or multi#professional) N2 (therap* or restor* or care* or team* or rehab*) ) OR AB ( (multi#disciplinary or inter#disciplinary or integrated or multi#modal or multi#professional) N2 (therap* or restor* or care* or team* or rehab*) ) 16,025

S12 S10 OR S11 39,619

S13 S9 AND S12 139

# Database: Svemed+ (Karolinska institutet)

1 exp:"Multiple Trauma" 110

2 exp:"Trauma, Nervous System" 1171

3 #1 OR #2 1275

4 exp:"Rehabilitation" 8299

5 #3 AND #4 243

6 exp:"Patient Care Team" 1242

7 #5 AND #6 14

Electronic search 05.07.19

# Database: Ovid MEDLINE(R) and Epub Ahead of Print, In-Process & Other Non-Indexed Citations and Daily <1946 to July 03, 2019>

5 July 2019

1 exp Multiple Trauma/ (12487)

2 exp Trauma, Nervous System/ (199254)

3 (multiple trauma or polytrauma or multitrauma or multi-trauma or (trauma* adj3 (spinal cord or nervous system or brain or amputation*))).ti,ab,kw. (48248)

4 1 or 2 or 3 (226263)

5 exp REHABILITATION/ (288696)

6 rehabilitation.fs. (190975)

7 rehabilitation.ti,ab,kw. (152174)

8 5 or 6 or 7 (492443)

9 (early or acute or fast track).ti,ab,kw. (2422834)

10 4 and 8 and 9 (4076)

11 exp Patient Care Team/ (65804)

12 ((multi?disciplinary or inter?disciplinary or integrated or multi?modal or multi?professional) adj3 (therap* or restor* or care* or team* or rehab*)).ab,ti,kw. (48082)

13 11 or 12 (106018)

14 10 and 13 (239)

# Database: Embase (Ovid) <1974 to 2019 July 03>

# 5 July 2019

1 multiple trauma/ (13903)

2 exp nervous system injury/ (306726)

3 (multiple trauma or polytrauma or multitrauma or multi-trauma or (trauma* adj3 (spinal cord or nervous system or brain or amputation*))).ti,ab,kw. (70778)

4 1 or 2 or 3 (330839)

5 exp rehabilitation/ (365070)

6 rh.fs. (145510)

7 rehabilitation.ti,ab,kw. (218187)

8 5 or 6 or 7 (554188)

9 (early or acute or fast track).ti,ab,kw. (3270367)

10 4 and 8 and 9 (7550)

11 ((multi?disciplinary or inter?disciplinary or integrated or multi?modal or multi?professional) adj3 (therap* or restor* or care* or team* or rehab*)).ab,ti,kw. (79545)

12 10 and 11 (336)

Cochrane library (Wiley) 5 July 2019

#1 MeSH descriptor: [Multiple Trauma] explode all trees 220

#2 MeSH descriptor: [Trauma, Nervous System] explode all trees 4601

#3 "multiple trauma" or polytrauma or multitrauma or multi-trauma or "multi trauma":ti,ab,kw (Word variations have been searched) 684

#4 trauma* near/3 ("spinal cord" or "nervous system" or brain or amputation*):ti,ab,kw (Word variations have been searched) 3782

#5 #1 or #2 or #3 or #4 7614

#6 MeSH descriptor: [Rehabilitation] explode all trees 32233

#7 rehabilitation:ti,ab,kw (Word variations have been searched) 43856

#8 #6 or #7 65156

#9 early or acute or "fast track":ti,ab,kw (Word variations have been searched) 228470

#10 #5 and #8 and #9 367

#11 (multi-disciplinary or multidisciplinary or inter-disciplinary or interdisciplinary or integrated or multi-modal or multimodal or multi-professional or multiprofessional) near/3 (therap* or restor* or care* or team* or rehab*):ti,ab,kw (Word variations have been searched) 9101

#12 #10 and #11 20

20 hits = 2 Cochrane reviews, 18 trials

# Cinahl (Ebsco) 1981-nå 5 July 2019

S1 (MH "Multiple Trauma") 2,977

S2 (MH "Spinal Cord Injuries+") OR (MH "Head Injuries+") OR (MH "Amputation, Traumatic") OR (MH "Spinal Injuries+") 63,843

S3 TI ( multiple trauma or polytrauma or multitrauma or multi-trauma or (trauma* N2 (spinal cord or nervous system or brain or amputation*)) ) OR AB ( multiple trauma or polytrauma or multitrauma or multi-trauma or (trauma* N2 (spinal cord or nervous system or brain or amputation*)) ) 18,170

S4 S1 OR S2 OR S3 70,987

S5 (MH "Rehabilitation+") 251,705

S6 TI Rehabilitation OR AB Rehabilitation 75,753

S7 S5 OR S6 293,854

S8 TI ( early or acute or "fast track" ) OR AB ( early or acute or "fast track" ) 419,957

S9 S4 AND S7 AND S8 2,257

S10 (MH "Multidisciplinary Care Team+") 38,879

S11 TI ( (multi#disciplinary or inter#disciplinary or integrated or multi#modal or multi#professional) N2 (therap* or restor* or care* or team* or rehab*) ) OR AB ( (multi#disciplinary or inter#disciplinary or integrated or multi#modal or multi#professional) N2 (therap* or restor* or care* or team* or rehab*) ) 24,640

S12 S10 OR S11 56,867

S13 S9 AND S12 157

# Svemed+ (Karolinska institutet) 5 July 2019

1 exp:"Multiple Trauma" 111

2 exp:"Trauma, Nervous System" 1197

3 #1 OR #2 1302

4 exp:"Rehabilitation" 8551

5 #3 AND #4 248

6 exp:"Patient Care Team" 1297

7 #5 AND #6 14

# Clinicaltrials.gov: 5 July 2019

Condition: (multiple trauma OR polytrauma OR multitrauma OR multi-trauma)
Other terms: (early OR acute OR “fast track”) AND rehabilitation 9 hits

# WHO: ICTRP 5 July 2019

Search 1:

Advanced search, in title: (“multiple trauma” OR polytrauma OR multitrauma OR multi-trauma) AND (early OR acute OR “fast track”) AND rehabilitation
recruitment status: All. 1 hit

Search 2:
Condition: “multiple trauma” OR polytrauma OR multitrauma OR multi-trauma (without synonyms)
Intervention: "early rehabilitation" OR "acute rehabilitation" OR “fast track” (without synonyms)
Recruiting status_ ALL

1 record : same as in the first search

**Supplemental Appendix S2** Characteristics of included studies

**Andelic et al. 2012**

| **Study / design**  (Study year, country, design, setting, duration, follow-up) | Design: Prospective cohort study  Setting: Level 1 trauma center  Country: Norway  Duration: 2 years (2005-2007)  Follow-up: 6 weeks and 12 months post-injury | |
| --- | --- | --- |
| **Participants**  (type of participants, total number, comparison group) | Men and women aged 16-55 years, admitted with ICD-10 diagnosis S06.0 – S06.9 within 24 h of injury, GCS less than 9, in need of neurointensive care for at least 5 days and survived the first year after injury.  Capacity (available bed) determined the assignment - whether the patients were admitted to the early rehabilitation section of the intensive care unit (intervention) or not (comparison group).  Total number: 31 in intervention group, 30 in control group  Mean age (SD) 29.4 (11.4) years | |
| **Interventions**  (what type of intervention, control group) | The intervention group received early comprehensive rehabilitation during acute TBI performed by an interdisciplinary rehabilitation team integrated in the acute care. The patients were directly transferred to a specialized rehabilitation center when medically stable.  The control group received either inpatient brain injury rehabilitation in sub-acute rehabilitation departments after a waiting period at a local hospital or nursing home or received no inpatient rehabilitation. | |
| **Outcomes** | Primary outcome; Glasgow outcome scale extended (GOSE) 12 months post-injury.  Secondary outcome: Disability Rating Scale (DRS), employment status and living status 12 months post-injury. | |
| **Risk of bias** | **Authors` judgement** | **Support for judgement** |
| *Random sequence generation (selection bias)* | **High risk** | No randomization. Age-selected cohort study |
| *Allocation concealment (selection bias)* | **High risk** | No randomization, available bed principle used |
| *Blinding of participants and personnel (performance bias)* | **High risk** | No blinding. Author performed outcome measures |
| *Blinding of outcome assessment (detection bias)* | **High risk** | No blinding, influence. Author performed outcome measures |
| *Incomplete outcome data (attrition bias) Objective outcome* | **Low risk** | Few missing data. Reasons unrelated to outcome. |
| *Incomplete outcome data (attrition bias) Subjective outcome* | **Low risk** | Few missing data. Reasons unrelated to outcome. |
| *Selective reporting (reporting bias)* | **Low risk** | All selected outcomes included |
| *Other bias* | **Low risk** | No other apparent sources of bias |

**Andelic et al. 2014**

| **Study / design**  (Study year, country, design, setting, duration, follow-up) | Design: Prospective cohort study. Same study population as Andelic et al 2012.  Setting: Level 1 trauma center  Country: Norway  Duration: 2 years (2005-2007)  Follow-up: 5 years post-injury | |
| --- | --- | --- |
| **Participants**  (type of participants, total number, comparison group) | Men and women aged 16-55 years, admitted with ICD-10 diagnosis S06.0 – S06.9 within 24 h of injury, GCS less than 9, in need of neurointensive care for at least 5 days and survived 5 years post-injury.  Capacity (available bed) determined the assignment - whether the patients admitted to the early rehabilitation section of the intensive care unit (intervention) or not (comparison group).  Total number: 59, 30 patients with continuous chain of treatment and 29 patients with broken chain of treatment  Mean age (SD) 29.4 (11.4) years (same as Andelic 2012) | |
| **Interventions**  (what type of intervention, control group) | The intervention group received early comprehensive rehabilitation during acute TBI performed by an interdisciplinary rehabilitation team integrated in the acute care. The patients were directly transferred to specialized rehabilitation center when medically stable.  The control group received either inpatient brain injury rehabilitation in sub-acute rehabilitation departments after a waiting period at a local hospital or nursing home or received no inpatient rehabilitation at all. | |
| **Outcomes** | Total rehabilitation costs at 1 year and 5 year post-injury  Disability Rating Scale at 6 weeks, 1 year and 5 years | |
| **Risk of bias** | **Authors` judgement** | **Support for judgement** |
| *Random sequence generation (selection bias)* | **High risk** | No randomization. Age-selected cohort study |
| *Allocation concealment (selection bias)* | **High risk** | No randomization, available bed principle used |
| *Blinding of participants and personnel (performance bias)* | **High risk** | No blinding. Author performed outcome measures |
| *Blinding of outcome assessment (detection bias)* | **High risk** | No blinding, influence. Author performed outcome measures |
| *Incomplete outcome data (attrition bias) Objective outcome* | **Low risk** | No missing data |
| *Incomplete outcome data (attrition bias) Subjective outcome* | **Low risk** | No missing data |
| *Selective reporting (reporting bias)* | **Low risk** | All selected outcomes included |
| *Other bias* | **Low risk** | No other apparent sources of bias |

**Ghaffar et al. 2006**

| **Study / design**  (Study year, country, design, setting, duration, follow-up) | Design: randomized clinical trial  Setting: two tertiary trauma centers  Country; Canada, Toronto  Duration: 18 months  Follow-up: 6 month post-injury | |
| --- | --- | --- |
| **Participants**  (type of participants, total number, comparison group) | Men and women 16-60 years with mild traumatic brain injury according to criteria of the American Congress of Rehabilitation Medicine.  Total number: 191, 97 in intervention group, 94 in control group  Mean age (SD): treated 30.7 (10.9) years, nontreated 33.3 (12.4) years | |
| **Interventions**  (what type of intervention, control group) | Intervention: treatment in a multidisciplinary TBI clinic within 1 week of injury. Treatments were tailored according to each individual patients need and included pharmacotherapy, supportive psychotherapy, physiotherapy and occupational therapy.  Control group: no follow-up | |
| **Outcomes** | 1. Symptoms of Post Concussion Disease ( Rivermead Post Concussion Disorder Questionaire (RPCQ)). 2. Psychosocial outcome (Rivermead Follow-up Questionnaire (RFQ)) 3. Subjective complaints of psychological distress (General Health Questionnaire (GHQ)) 4. Cognitive outcome (The Stroop Color – Word Test, The Symbol Digit Modalities test, The Paced Visual Serial addition task, Simple Reaction time, Choice Reaction Time) 5. Memory and executive function (The Hopkins Verbal Learning Test, The Vocabulary subtest of the Wechsler Adult Intelligence scale, The Letter-Number Sequencing subtest of the WAIS-III and The Matrix-Reasoning subtest of the WAIS-III) | |
| **Risk of bias** | **Authors` judgement** | **Support for judgement** |
| *Random sequence generation (selection bias)* | **Low risk** | Randomization, unclear procedure |
| *Allocation concealment (selection bias)* | **Unclear risk** | No information |
| *Blinding of participants and personnel (performance bias)* | **High risk** | No blinding |
| *Blinding of outcome assessment (detection bias)* | **Unclear risk** | Some data are no blinding and no influence (low risk), but unclear if all data are with no influence |
| *Incomplete outcome data (attrition bias) Objective outcome* | **High risk** | Missing data are unbalanced |
| *Incomplete outcome data (attrition bias) Subjective outcome* | **High risk** | Missing data are unbalanced |
| *Selective reporting (reporting bias)* | **Low risk** | All expected data are included |
| *Other bias* | **Low risk** | No other apparent sources of bias |

**Lui et al. 2014**

| **Study / design**  (Study year, country, design, setting, duration, follow-up) | Design: retrospective cohort study. A pilot study.  Setting: neurosurgical department of a tertiary hospital  Duration: 15 months (nov 2010 – feb 2012)  Country; Singapore  Follow-up: none | |
| --- | --- | --- |
| **Participants**  (type of participants, total number, comparison group) | All patients with TBI, 17% moderate to severe TBI  Total number : 119  Intervention group: 68  Historical/Control group: 51  Mean age (SD): 61.8 (19.1) years | |
| **Interventions**  (what type of intervention, control group) | Intervention: very early integrated TBI rehabilitation; twice weekly multidisciplinary reviews from a rehabilitation team while still in the acute unit  Control group: historical data | |
| **Outcomes** | 1. Functional outcome (FIM) 2. Length of stay (acute and total) 3. Surgical interventions 4. Medical complications | |
| **Risk of bias** | **Authors` judgement** | **Support for judgement** |
| *Random sequence generation (selection bias)* | **High risk** | Date of admission, judgement of the clinician |
| *Allocation concealment (selection bias)* | **High risk** | Data of admission |
| *Blinding of participants and personnel (performance bias)* | **High risk** | No blinding |
| *Blinding of outcome assessment (detection bias)* | **High risk** | No blinding, influence |
| *Incomplete outcome data (attrition bias) Objective outcome* | **Low risk** | No blinding, no influence |
| *Incomplete outcome data (attrition bias) Subjective outcome* | **Low risk** | Reasons unrelated to outcome |
| *Selective reporting (reporting bias)* | **Low risk** | All expected outcomes included |
| *Other bias* | **Low risk** | No other apparent sources of bias |

**Mackay et al. 1992**

| **Study / design**  (Study year, country, design, setting, duration, follow-up) | Design: retrospective cohort study  Setting: patients discharged from an inpatient rehabilitation facility – historical data  Country: USA, Hartford, Connecticut  Duration: Sept 1984 – May 1990  Follow-up: none | |
| --- | --- | --- |
| **Participants**  (type of participants, total number, comparison group) | Patients with severe TBI, men and women, admitted to a trauma center and who received inpatient rehabilitation.  Total number: 38. 17 in intervention group, 21 in comparison group  Mean age (SD): treated 29.1(3.2) years, nontreated 30.0 (2.2) years | |
| **Interventions**  (what type of intervention, control group) | Intervention: acute services with formalized early intervention TBI program  Control group: acute care without formalized TBI program. | |
| **Outcomes** | Measured at discharge from rehabilitation facility:   1. Physical and motor skills 2. Sensory and perceptual skills 3. Cognitive and language skills   Other outcomes:   - length of coma - RLA at discharge from acute facility - RLA at discharge from rehabilitation facility - Disposition at discharge (home vs extended care facility) - Length of stay in the acute hospital - Length of stay in the rehabilitation facility | |
| **Risk of bias** | **Authors` judgement** | **Support for judgement** |
| *Random sequence generation (selection bias)* | **High risk** | No randomization, place of admission |
| *Allocation concealment (selection bias)* | **High risk** | Place of admission |
| *Blinding of participants and personnel (performance bias)* | **High risk** | No blinding |
| *Blinding of outcome assessment (detection bias)* | **Unclear risk** | Some outcomes are no blinding and no influence. Unclear if all data are without influence |
| *Incomplete outcome data (attrition bias) Objective outcome* | **Low risk** | Missing data unrelated to outcome |
| *Incomplete outcome data (attrition bias) Subjective outcome* | **Low risk** | Missing data unrelated to outcome |
| *Selective reporting (reporting bias)* | **Low risk** | All expected outcomes included |
| *Other bias* | **High risk** | Less than 25 participants in each group |

**Supplemental Appendix S3** Early interdisciplinary rehabilitation, 2. screening

| **Author** | **Title** | **Year** | **Participants trauma ≥ 18 years** | **Intervention**  **EIR** | **Comparison**  **CAU** | **Outcomes pre-defined for this review** | **I/E** |
| --- | --- | --- | --- | --- | --- | --- | --- |
| Turner-Stokes et al. | Multi-disciplinary rehabilitation for acquired brain injury in adults of working age | 2015 | Yes | Uncertain | Uncertain |  | E |
| Singh et al. | Clinical pathways in head injury; improving the quality of care with early rehabilitation | 2012 | Yes | Yes | No |  | E |
| Wang et al. | Rehabilitation practice and outcomes after spinal cord injury | 2015 |  |  |  |  | *E*  *Did not find article* |
| Gentleman | Rehabilitation after traumatic brain injury | 2001 | Yes | No | No |  | E |
| Emerich et al. | Competent care for persons with spinal cord injury and dysfunction in acute inpatient rehabilitation | 2012 | Yes | No | No |  | E |
| Sörbo et al | Well-integrated chain of care results in better prognosis in severe brain injury | 2001 | Yes | Yes | No | Integrated chain of care, not a trial | E |
| Vanderploeg et al. | Rehabilitation of traumatic brain injury in active duty military personnel and veterans; defense and veterans brain injury center randomized controlled trial of two rehabilitation approaches | 2008 | Yes | Yes | No |  | E |
| Mckay | Benefits of a formalized traumatic brain injury program within a trauma center | 1994 | Yes | No | No |  | E |
| Taheri et al. | Physician resource utilization after geriatric trauma | 1997 | yes | Yes | No |  | E (resolved by discussion) |
| Andelic et al. | Does an early onset and continuous chain of rehabilitation improve the long-term functional outcome of patients with severe traumatic brain injury? | 2012 | Yes | Yes | Yes | GOSE, disability rating scale (DRS) | I |
| Saunders | Preventing secondary complications in trauma patients with implementation of a multidisciplinary mobilization team | 2015 | Yees | Yes | No |  | E |
| Hall et al. | Multidisciplinary approaches to management of acute head injury | 1992 | Yes | yes | No |  | E (resolved by discussion) |
| Lippert- Gruner et al. | Early stimulation, a component of an early rehabilitation treatment concept on the neurosurgical intensive care unit | 1997 | Yes | Yes | No |  | E (resolved by discussion) |
| Bartolo et al. | Early rehabilitation for severe acquired brain injury in intensive care unit; multicenter observational study | 2016 | Yes | Yes | No |  | E |
| Ayvazian et al. | Clinical management of veterans with traumatic brain injury within the context of polytrauma |  | Yes | No | Yes |  | E |
| Bouman et al. | Effects of an integrated 'Fast Track' rehabilitation service for multi-trauma patients: Non-randomized clinical trial | 2017 | Yes | ? | Yes | FIM, short form SF-36, MMSE, HADS | E (resolved by discussion, including third reviewer) |
| Choi et al. | Multimodal early rehabilitation and predictors of outcome in survivors of severe traumatic brain injury | 2008 | Yes | Yes | No |  | E |
| De Guise et al. | Effect of an integrated reality orientation programme in acute care on post-traumatic amnesia in patients with traumatic brain injury | 2005 | Yes | No | Yes |  | E (resolved by discussion) |
| Gruner et al. | Multimodal early onset stimulation (MEOS) in rehabilitation after brain injury | 2000 | Yes | Yes | No |  | E |
| Katz et al | Recovery of ambulation after traumatic brain injury | 2004 | Yes | No | Yes |  | E |
| Kosar et al. | Cost-effectiveness of an integrated fast track rehabilitation service for multi-trauma patients | 2009 | Yes | Yes | Yes |  | E  Protocol, Bouman et al. 2017 |
| Leng et al. | The traumatic brain injury early rehabilitation specialized program |  | Yes | Yes | Yes | LOS, FIM | E (resolved by discussion) |
| Lovio et al. | Acute and long-term neurorehabilitation; a comprehensive (follow-up) programme for children and young adults with acute brain injury of all severities |  | Yes | No | No |  | E |
| Lui et al. | A pilot project of early integrated traumatic brain injury rehabilitation | 2014 | Yes | Yes | Yes | FIM, ALOS, RLOS, total LOS, complications, surgical interventions | I |
| Middleton et al. | Right care, right time, right place: improving outcomes for people with spinal cord injury through early access to intervention and improved access to specialised care: study protocol | 2014 | Yes | No | No |  | E |
| Nordin | *Gravt hjärnskadade; tidlig stimulering effektiv* (Swedish) | 1999 | Yes | No |  |  | E |
| Pignat et al. | From disorders of consciousness to early neurorehabilitation using assistive technologies in patients with severe brain damage | 2015 | No | Yes | Yes |  | E |
| Seel et al. | Specialized early treatment for persons with disorders of consciousness; program components and outcomes | 2013 | Yes | Yes | No |  | E |
| Seeto et al. | Feasibility of an interdisciplinary early intervention for patients with low levels of responsiveness following an acquired brain injury | 2013 | Yes | No | Yes |  | E (resolved by discussion) |
| Stanley et al. | Outcomes for patients with traumatic brain injury utilizing continuum of care model |  | Yes | Yes | No |  | E |
| Turner-Stokes | Evidence for the effectiveness of multi-disciplinary rehabilitation following acquired brain injury; a synthesis of two systematic approaches | 2008 | Yes | Yes | No |  | E |
| Turner-Stokes et al. | Cost-efficiency of specialist hyperacute in-patient rehabilitation services for medically unstable patients with complex rehabilitation needs: a prospective cohort analysis | 2016 | Yes | Yes | No |  | E (resolved by discussion) |
| Wijnen et al | Cost-effectivness of an integrated “fast track” rehabilitation service for multitrauma patients; Non-randomized clinical trial |  | Yes | No | Yes |  | E |
| von Wild | Early neurosurgical rehabilitation of cranial cerebral injured patients. 1. A new conception |  | Yes | Yes | No |  | E |
| Sörbo et al. | *Sammanhållen vårdkedja ger bättre prognos vid svår hjernskada* (Swedish) | 2001 | yes | No | No |  | E |
| Ghaffar et al. | Randomized treatment trial in mild traumatic brain injury | 2006 | Yes | Yes | Yes | physician visits post-MTBI, GHQ, RPCQ, Stroop, WAIS, Hopkins verbal learning | I |
| Elgmark et al. | Mild traumatic brain injuries; the impact of early intervention on late sequelae. Randomized controlled trial | 2007 | Yes | No | Yes |  | E |
| Paniak et al. | A randomized trial of two treatments for mild traumatic brain injury | 1998 | Yes | No | Yes |  | E |
| Salazar et al. | Cognitive rehabilitation for traumatic brain injury. Randomized trial | 2000 | Yes | No | No |  | E |
| Wade et al. | Does routine follow up after head injury help? Randomized controlled trial | 1998 | Yes | No |  |  | E |
| Shiel et al. | The effects of increased rehabilitation therapy after brain injury; results of a prospective controlled trial | 1999 | Yes | Yes | No |  | E |
| Slade et al. | Randomized controlled trial to determine the effect of intensity of therapy upon length of stay in a neurological rehabilitation setting | 2002 | No/yes | Yes | No |  | E |
| Zhu et al. | Does intensive rehabilitation improve the functional outcome of patients with traumatic brain injury? Randomized controlled trial | 2007 | Yes | No | No |  | E |
| Mackay et al. | Early intervention in severe head injury; long-term benefits of a formalized program | 1992 | Yes | Yes | Yes |  | I |
| Andelic et al. | Cost-effectiveness analysis of an early-initiated, continuous chain of rehabilitation after severe traumatic brain injury | 2014 | Yes | Yes | Yes | hospitalization costs, incremental cost-effectivness ratios (ICER), disability rating scale (DRS) | I |

I; included, E; excluded. Green color; article included by both reviewer. Orange color; article excluded by both reviewer. Yellow color; disagreement, resoled by discussion.

**Supplemental Appendix S4** Comparison EIR versus control.

Primary outcomes, Outcome 1; functional outcome

| **Functional outcome** | **Time measured post-injury** | **Intervention group N (%)** | **Control group**  **N (%)** | **Statistical method** | **Effect size** | **Comment** |
| --- | --- | --- | --- | --- | --- | --- |
| GOSE  (Andelic 2012)  DRS  (Andelic 2012)  DRS  (Andelic 2014)  FIM  (Lui 2014)  RLA acute  (Mackay 1992)  RLA rehab  (Mackay 1992) | 12 months  12 months  5 years  FIM gain during inpatient rehabilitation stay  Discharge from acute ward  Discharge from rehab | 31/31 (100)  31/31 (100)  30/30 (100)  68 (100)  17/17 (100)  17/17 (100) | 30/30 (100)  30/30 (100)  29/29(100)  51 (100)  21/21(100)  21/21(100) | Proportional OR unadjusted (95% CI*) p-value*  T-test  Means of AUC analyses  Regression analysis  *t-test*  *t-test* | 3.25 (1.08 – 9.87)  *P=0.03*  p= 0.03  Intervention: 19.40. SD: 26.60  Control: 23.46. SD:22.91  *p=* 0.201  p=0.003  p=0.05 | small number of patients  High SD  Mean age in intervention group was 66.2±17.0, for control group (historical data) the mean age was 47.8±20.1. p˂0.001.  Factors associated with higher FIM gain was shorter RLOS and younger age. Lower FIM was associated with higher FIM score at admission  Small groups  Small groups |

GOSE;Glasgow Outcome Scale Extended, FIM; functional independency measures, DRS; Disability Rating Scale, RLA; Rancho Los Amigos Scale of Cognitive Functioning, NS: no statistical significance

Comparison EIR versus control. Primary outcome, Outcome 2; participation

| **Participation** | **Time measured post injury** | **Intervention group N (%)** | **Control group**  **N (%)** | **Statistical method** | **Effect size** | **Comments** |
| --- | --- | --- | --- | --- | --- | --- |
| Return to work  (Andelic 2012) | 12 months | 31/31 (100) | 30/30 (100) |  | NS | 39% returned to work in intervention group vs 27% in control group |

NS: no statistical significance

Comparison EIR versus control. Secondary outcome; Outcome 3; Length of stay

| **Length of stay** | **Intervention group N (%)** | **Control group**  **N (%)** | **Statistical method** | **Effect size** | **Comment** |
| --- | --- | --- | --- | --- | --- |
| LOS ICU  (Andelic 2012)  LOS total  (Andelic 2012)  LOS total  (Lui 2014)  ALOS (days)  (Lui 2014)  RLOS (days)  (Lui 2014)  LOS total  (Mackay 1995)  ALOS  (Mackay 1995)  RLOS  (Mackay 1995) | 31/31 (100)  31/31 (100)  68/68 (100)  68/68 (100)  68/68 (100)    17/17 (100)  17/17 (100)  17/17 (100) | 30/30 (100)    30/30 (100)  51/51 (100)  51/51 (100)  51/51 (100)  21/21 (100)  21/21 (100)  21/21 (100) | Mann-Whitney U test  Mann-Whitney U test  Spearmans rank correlation coefficient  Spearmans rank correlation coefficient  Spearmans rank correlation coefficient  *t-test*  *t-test*  *t-test* | *P=*0.34  *P=*0.07  *p=*0.329  *p=0.929*  *p=0.089*  *p=0.028*  *NS*  *P=0.026* | For intervention group 51.5±6.8, for control group 4.1±7.8 |

LOS; length of stay, ICU; intensive care unit , RLOS; rehabilitation length of stay, ALOS; acute length of stay. NS; no statistical significance

Comparison EIR versus control. Secondary outcome; Outcome 5; Complications during hospital stay

| **Complications** | **Intervention**  **N (%)** | **Control**  **N (%)** | **Statistical method** | **Effect size** | **Comments** |
| --- | --- | --- | --- | --- | --- |
| Complications  (Lui 2014) | 68/68 (100) | 51/51 (100) | unclear | NS | Rate of medical complications was 39.7% in the intervention group and 37.3% in control group.  Heterogenous groups (intervention vs control/historical data), wide spectrum of injury severity (all severities of TBI included). |

NS: non significance

Comparison EIR versus control. Secondary outcome; Outcome 8; Socioeconomic costs

| **Socioeconomic costs** | **Intervention**  **N (%)** | **Control**  **N (%)** | **Statistical method** | **Effect size** | **Comments** |
| --- | --- | --- | --- | --- | --- |
| Total hospitalization costs at 1 year  (Andelic 2014)  Total hospitalization costs at 5 years  (Andelic 2014) | 30/30 (100)  30/30 (100) | 29/29 (100)  29/29 (100) | ICER  ICER | Dominant  Dominant | Few participants. Uncertain effect using DRS.  Few participants. Uncertain effect using DRS |

ICER; incremental cost-effectiveness ratio: ICER= delta kostnad/delta effect
